# Demographic and mental health characteristics of individuals in the NSW Housing and Accommodation Support Initiative (HASI), Community Living Supports and HASI Plus

**DOI:** 10.1177/10398562251316431

**Published:** 2025-01-30

**Authors:** Gary KK Low, Jason Li, Emily Hielscher, Veronica Sheanoda, Sumathi Govindasamy, Fadzi Marasha

**Affiliations:** Sydney Medical School, Faculty of Medicine and Health, University of Sydney, Sydney, NSW, Australia; Outcome and Improvement Team, Flourish Australia, Olympic Park, NSW, Australia; and Nepean Blue Mountain Local Health District, Penrith, NSW, Australia; Sydney School of Public Health, Faculty of Medicine and Health, University of Sydney, Sydney, NSW, Australia; Outcome and Improvement Team, Flourish Australia, Olympic Park, NSW, Australia

**Keywords:** mental health, recovery, RAS, CANSAS

## Abstract

**Objective:**

To investigate the demographic characteristics associated with mental health recovery measures among individuals accessing the Housing and Accommodation Support Initiative (HASI) program over a 19-year period.

**Methods:**

This was a retrospective cohort study conducted from January 2004 to October 2023. The Camberwell Assessment of Need Short Appraisal Schedule (CANSAS) and Recovery Assessment Scale (RAS) were used as measures of mental health recovery.

**Findings:**

A total of 2350 people with an average age of 42 years old were included. Female accounts for 46.0% of the total. The proportion of unmet needs in the CANSAS reduced from a median of 33.3% of the first follow-up to 5.8% in the 20^th^ follow-up. The average RAS scores were above three, indicating agree and strongly agree in all domains and improved in each follow-up. First Nations were associated with higher unmet needs in ‘psychotic symptoms’, ‘safety to others’ and ‘transport’ CANSAS domains, and LGBTI had reduced RAS scores in all domains.

**Conclusion:**

HASI program engagement is associated with the reduction of unmet needs and improvement of the recovery of individuals with severe mental illness. Age, sex, gender, LGBTI, First Nations and country of birth were associated with changes in the CANSAS and RAS outcomes.

Community Living Supports (CLS), Housing and Accommodation Support Initiative (HASI) and HASI Plus (collectively called the ‘HASI program’ in this study) are funded initiatives by the New South Wales (NSW) Ministry of Health. The HASI and CLS programs serve as the foundation for the NSW government mental health reformation with the key objective to prevent unnecessary hospitalisations and emergency department presentation due to mental health. The eligibility for HASI programs is persons aged 16 years or older, have a diagnosis of a mental illness which causes difficulties in day-to-day life and who wants to receive help to develop and achieve goals.^1^

HASI and CLS deliver community-based psychosocial support through Community Managed Organisations (CMOs) and Local Health District. Community-based psychosocial support sand HASI Plus provides fit for purpose community-based accommodation, flexible intense accommodation support, high intensity psychosocial support and multidisciplinary mental health support.^2–5^ The HASI program was evaluated in 2022 on a service level by the Social Policy Research Centre, University of New South Wales.^4^ Similar to mentally supported accommodation services internationally, there is an effect in reducing hospital admissions, reduced duration of hospital stay and increased uptake of appropriate mental health services.

To our knowledge, no evaluation of the program from an individual level has been studied. Individual data may reveal associations between demographic characteristics and mental health recovery outcomes from HASI engagement. An analysis taken over the study period will reveal gaps and areas to improve, enabling specialisation and targeting in respect to priority populations. Hence, this study aimed to investigate the demographic characteristics associated with mental health recovery measures among individuals accessing the HASI program over a 19-year period.

## Methods

This is a retrospective cohort study on all individuals in the HASI program supported by Flourish Australia between January 2004 and October 2023. People that had relocated HASI sites or had re-engaged after exiting from HASI program were also included in the analysis.

The peer workers assessed and followed up with all eligible individuals in the HASI program and these were recorded in the Flourish Australia database. The demographic variables included in this study were age, number of years in HASI program, sex, gender identity, LGBTI status, marital status, First Nations and country of birth. Age is defined as the age in years at the start of HASI program engagement. Sex variable refers to biological sex, gender identity is person’s self-concept of their gender and LGBTI status represents one’s sexual orientation.^6^ ‘Not stated/unknown/not known’ in gender identity and marital status was deemed as missing value and was excluded from the analysis. First Nations included Aboriginal and/or Torres Strait Islander. ‘Australia’ and ‘outside Australia’ were defined according to the country of birth.

### Outcome measures

Outcomes to be assessed were in the domains of psychosocial need and recovery measured by the validated questionnaires: Camberwell Assessment of Need Short Appraisal Schedule (CANSAS)^7^ and Recovery Assessment Scale (RAS),^8^ respectively. CANSAS was first introduced to the HASI programs in March 2008, followed by RAS in March 2011. Hence, the outcomes were assessed according to these periods onwards. All outcomes were compared among the follow-ups to reflect the change of outcome during the HASI program enrolment. ‘Follow-ups’ is defined as the sequence of assessments with CANSAS and RAS conducted by the individuals, and it is denoted by numbers. The median interval of each CANSAS follow-up was 5.8 months with interquartile range (IQR) of 4.1-8.3 months. The median interval of each RAS follow-up was 3.9 months (IQR: 2.3-6.1 months).

### CANSAS

In Australia, CANSAS is the most widely used outcome tool for needs assessment and predominantly used by CMOs. The CANSAS has a high degree of inter-rater and test-retest reliability and is valid in terms of content and face validity.^9^ CANSAS outcome was summarised by the percentage of unmet needs. Additionally, each question of CANSAS, also known as domain, was described. There were 22 domains with three possible answers: ‘no serious problem’, ‘no serious problem or moderate problem because of continuing intervention (met need)’ and ‘current serious problem (unmet need)’.^7^

### RAS

RAS is an internationally widely used recovery assessment scale.^10^ To measure recovery the RAS scale was adopted, a 41-item measure with key domains of personal confidence and hope, willingness to ask for help, goal and success orientation, reliance on others and not dominated by symptoms. Items pertaining to these domains were rated using a 5-point scale from 1 strongly disagree to 5 strongly agree.^11^ Construct validity, test-retest and inter-rater reliability were deemed satisfactory.^8^

### Statistical analysis

The study time for each participant is structured as the total time period from first entry into the HASI program to the date of last recorded exit. Participants with multiple exits from the program were hence counted for the entire study period until last recorded exit. All missing data were excluded from the analysis.

CANSAS was described by summarising responses into median of proportion of unmet needs which is defined as the median of all unmet needs in a follow-up period divided by the total number of needs. A time series analysis was performed on the median of proportion of unmet needs with the assumption that the follow-ups are conducted 6-monthly. Decomposition of the additive time series was performed to evaluate the seasonal, trend and random effect. Decomposition of the time series was performed to determine if data was subject to seasonality or other repeating long-term predictable patterns that may obscure effect size. Univariable and multivariable Poisson model of generalised estimating equations was used to analyse the proportion of unmet needs in each follow-up as the dependent variable and the demographic characteristics as the independent variables. Similarly, binomial model of generalised estimating equations was used to analyse each of the domain with met/unmet needs as the dependent variable and demographic characteristics as dependent variables. Question 12, 13, 17 and 18 were not analysed due to diverging estimates (‘one or more categories has zero count’). These questions represent domains of ‘alcohol’, ‘drugs’, ‘dependents’ and ‘basic education’, respectively.

RAS was summarised by the five domains with a mean of five-point agreement scale with ‘1’ as strongly disagree and ‘5’ as strongly agree for each follow-up.^11^ A time series analysis was performed and decomposing the additive time series was performed for RAS. A linear mixed model was used to analyse the domains’ score as the dependent variable and the demographic characteristics as the independent variables. In both generalised estimating equations and linear mixed models, the random effect was the multiple follow-ups of an individual, and the fixed effect was the independent variables. R version 4.3.0 was used for the analysis.

This study was approved by the Nepean Blue Mountain Local Health District Low Risk Subcommittee (Ethics number: 2023/ETH02550). Participant’s informed consent was exempted.

## Result

A total of 2350 people in Flourish Australia were engaged in HASI program between 2004 and 2023. The number participants with multiple exits from the program were 346(14.7%). The average age when engaged at the start of HASI program was 42 years old with standard deviation (SD) of 12.4 years. The biological sex distribution for female was 1080 (46.0%). The demographic characteristics of the people in HASI program is tabulated in Table 1.

**Table 1.** Demographic characteristics of the people in HASI program

Characteristics	N = 2350
Age when HASI program started (n = 2348), mean (SD)	42 (13.4)
Age of death (n = 96), mean (SD)	55.1 (10.9)
Number of years in HASI program (n = 2021), median (IQR)	1.0 (0.4-2.4)
Sex (n = 2350), n (%)	
Female	1080 (46)
Gender identity (n = 1470), n (%)	
Female	646 (43.9)
Male	736 (50.1)
Non-binary	13 (0.9)
Transgender	7 (0.5)
Not stated/not known	68 (4.6)
LGBTI (n = 2260), n (%)	
Yes	85 (3.8)
Marital status (n = 1673), n (%)	
Single	1108 (66.2)
Divorced	121 (7.2)
Separated	97 (5.8)
Unmarried	77 (4.6)
Long-term relationship	76 (4.5)
Married	57 (3.4)
Widowed	32 (1.9)
Unknown	105 (6.3)
First Nations (n = 2322), n (%)	
First Nations	477 (20.5)
Non-First Nations	1845 (79.5)
Country of birth (n = 2342), n (%)	
Australia	2094 (89.4)
Outside Australia	248 (9.6)

The most common primary mental health diagnosis was schizophrenia with 821(49.1%), followed by bipolar affective disorder with 169(10.1%) and schizoaffective disorder with 155(9.3%). All the primary diagnoses are tabulated in Table 2.

**Table 2.** The proportion of primary diagnosis among the people in HASI program

Primary diagnosis	n(%)
Schizophrenia	821 (49.1)
Bipolar affective disorder	169 (10.1)
Schizoaffective disorder	155 (9.3)
Depressive symptoms	122 (7.3)
Personality disorder	84 (5)
Generalised anxiety disorder	80 (4.8)
Post-traumatic stress disorder	52 (3.1)
Major depressive disorder	47 (2.8)
Other	45 (2.7)
Anxiety disorders	42 (2.5)
Depressive disorder NOS	24 (1.4)
Mixed anxiety and depressive symptoms	11 (0.7)
Social phobia	4 (0.2)
Anxiety symptoms	4 (0.2)
Agoraphobia	3 (0.2)
Attention deficit hyperactivity disorder (ADHD)	2 (0.1)
Adjustment disorder	2 (0.1)
Acute distress disorder	1 (0.1)
Dysthmia	1 (0.1)
Brief psychotic disorder	1 (0.1)
Other psychotic disorder	1 (0.1)
Eating disorder	1 (0.1)
Total	1672 (100)

## CANSAS

**Figure 1.**
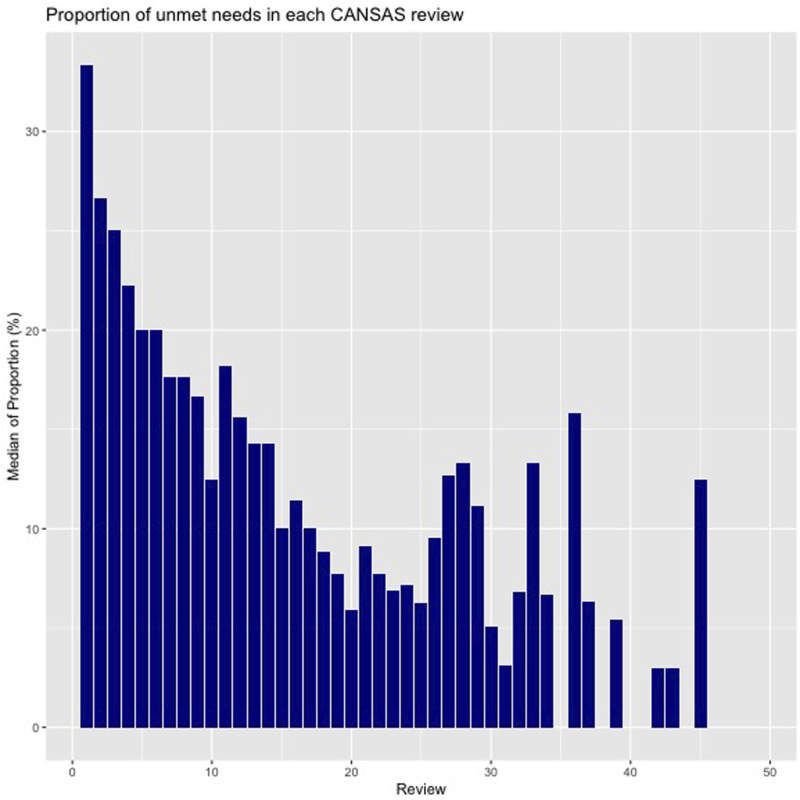
Proportion of unmet needs in each CANSAS review.

The proportion of unmet needs in the CANSAS reduced from a median of 33.3% (IQR: 12.5-60) of the first follow-up to 5.8% (IQR: 0-30.8) in the 20^th^ follow-up. Figure 1 displays the proportion of the unmet needs in each of the 51 follow-ups. The details of the proportion of unmet needs and total number of needs are tabulated in supplemental S1. The decomposition of the additive time series of the proportion of unmet needs indicated that the trend was unaffected by seasonality and random effect, that is, the observed effect is not affected by long-term predictable cyclical patterns or unexpected single events, hence producing similar trend line between observed and trend graphs in S2.

In the multivariable generalised estimating equations model, the overall proportion of unmet needs increased significantly with younger age and for First Nations, with adjusted odds ratio (OR) of 1.007 [95% confidence interval (CI): 1.003-1.011] and 1.17 [95% CI: 1.04-1.32], respectively. Conversely, the proportion of unmet needs was reduced for male gender with an adjusted OR of 0.70 [95% CI: 0.52-0.94]. Both the univariable and multivariable models’ coefficients are tabulated in Table 3.

**Table 3.** Univariable and multivariable generalised estimating equations of the proportion of unmet needs of CANSAS

	Univariable model	Multivariable model
	Coefficients (SE)	Coefficients (SE)
**Age**	−0.01 (0.001)**	−0.01 (0.002)**
**Sex: (Ref = female)**		
Male	−0.08 (0.04)	0.22 (0.15)
**Gender identity: (Ref = female)**		
Male	−0.15 (0.05)**	−0.35 (0.15)*
Non-binary	0.64 (0.17)**	0.36 (0.21)
Transgender	0.14 (0.28)	−0.11 (0.31)
**LGBTI: (Ref = No)**		
Yes	0.26 (0.09)**	0.16 (0.12)
**Marital status: (Ref = divorced)**		
Long-term relationship	0.12 (0.13)	−0.02 (0.13)
Married	−0.06 (0.13)	−0.07 (0.14)
Separated	0.20 (0.11)	0.13 (0.12)
Single	−0.02 (0.08)	−0.15 (0.08)
Unmarried	0.10 (0.12)	−0.02 (0.12)
Widowed	0.07 (0.15)	0.07 (0.15)
**First Nations: (Ref = no)**		
Yes	0.25 (0.05)**	0.16 (0.06)*
**Country of birth: (Ref = Australia)**		
Outside Australia	−0.26 (0.06)**	−0.14 (0.08)

Abbreviation: SE, standard error; Ref, reference group.

The intercepts coefficient for univariable model (in order from top to bottom of the table): 1.37, 1.03, 1.02, 0.97, 0.97, 0.93 and 1.01.

The intercept coefficient for multivariable model is 1.37.

*p-value <.05.

**p-value <.01.

### Multivariable models of each CANSAS domains

In the ‘accommodation’ domain, the age was statistically significant indicating the increased unmet needs with younger age with OR of 1.02 (95% CI: 1.0004-1.0404). In the ‘food’ domain, the statistically significant characteristics were male sex, male gender identity, non-binary gender identity, transgender gender identity and First Nations. Being a male has an OR of 2.89 (95% CI: 1.15-7.25). The models’ coefficients are tabulated in S4 and S5 and the OR of statistically significant demographic characteristics is tabulated in Table 4.

**Table 4.** Odds ratios of statistically significant demographic characteristics (*p* < .05) for the CANSAS domains

	Demographic characteristics										
CANSAS domains, OR (95% CI)	Age	Male sex	Male gender identity	Non-binary gender identity	Transgender identity	First Nations	Born outside Australia	Single	Long-term relationship	Married	Separated
Accommodation	1.02 (1.0004-1.0404)										
Food		2.89 (1.15-7.25)	0.31 (0.12-0.79)	0.23 (0.057-0.89)	9.87 (2.099-46.45)	1.46 (1.09-1.96)					
Looking after the home							0.57 (0.39-0.83)	0.65 (0.47-0.91)			
Self-care					4.71 (1.84-12.07)		0.58 (0.39 -0.89)				
Day time activities	1.01 (1.004-1.02)						0.73 (0.55-0.98)				
Psychotic symptoms						1.55 (1.18-2.04)					
Psychological distress						1.27 (1.005-1.61)					
Company				4.95 (1.93-12.7)							
Intimate relationships	0.99 (0.97-1)								0.29 (0.14-0.6)	0.21 (0.09-0.49)	
Sexual expression	0.99 (0.97-1)	3.03 (1.39-6.65)					0.56 (0.32-0.97)			0.35 (0.12-0.98)	0.32 (0.13-0.77)
Digital communication	0.98 (0.96-1)						0.54 (0.34-0.87)				
Transport						1.6 (1.23-2.11)					
Money	1.01 (1.002-1.018)										
Benefits	1.02 (1.012-1.028)										

Abbreviation: OR, odds ratio; CI, confidence interval.

Being single and born outside Australia were associated with fewer unmet needs in the ‘looking after the home’ domain. In ‘self-care’ domain, transgender and born outside Australia were statistically significant. Age and being born outside Australia in ‘daytime activities’ domain were statistically significant. The models’ coefficients are tabulated in supplemental tables S6-S8.

No characteristics were statistically significant in ‘physical health’, ‘information on condition and treatment’ and ‘safety to self’ domains. The models’ coefficients are tabulated in S9, S11 and S13. First Nations was statistically significant in ‘psychotic symptoms’, ‘psychological distress’ and ‘safety to others’ domains. The models’ coefficients are tabulated in S10, S12 and S14.

In ‘company’ domain, non-binary gender identity was statistically significant. In ‘intimate relationships’ domain, age, long-term relationship and being married were statistically significant. In ‘sexual expression’ domain, the statistically significant characteristics were age, male sex, being married, separated and born outside Australia. Age and born outside Australia were statistically significant in ‘digital communication’ domain. The models’ coefficients are tabulated in S15-S18. In ‘transport’ domain, First Nations was statistically significant. Age in ‘money’ domain and ‘benefits’ domain was statistically significant. The models’ coefficients are tabulated in S19-S21.

## RAS

A total of 139 follow-ups were conducted for RAS. The RAS consistently scored an average above three (i.e. responded with agree or strongly agree) in all domains for all follow-ups of all individuals (S22-26). Similarly, the decomposition of the additive time series of all the RAS domain scores indicated that the trend was unaffected by the seasonal and random components. The number of respondents in each follow-up of RAS is tabulated in S27.

In ‘personal confidence and hope’ domain, the multivariable linear mixed model yielded statistical significance for male sex, LGBTI, separated and being born outside Australia. In ‘willingness to ask for help’, age, male sex, male gender, LGBTI, separated and being born outside Australia were statistically significant. Male sex, LGBTI and being separated were the statistically significant characteristics for domain ‘goal and success orientation’. In ‘reliance on others’, only male sex was statistically significant. LGBTI and non-binary gender identity were statistically significant in domain ‘no domination by symptoms’. Table 5–9 tabulated the models’ coefficients for all five domains of RAS.

**Table 5.** Univariable and multivariable of linear mixed model of the domain ‘Personal confidence and hope’ of RAS

	Univariable model	Multivariable model
	Coefficients (SE)	Coefficients (SE)
**Age**	−0.0002 (0.001)	0.002 (0.002)
**Sex: (Ref = female)**		
Male	0.21 (0.03)	0.60 (0.30)*
**Gender identity: (Ref = female)**		
Male	0.25 (0.04)**	−0.35 (0.30)
Non-binary	−0.56 (0.19)**	−0.35 (0.21)
Transgender	0.14 (0.32)	0.39 (0.44)
**LGBTI: (Ref = no)**		
Yes	−0.40 (0.08)**	−0.34 (0.10)**
**Marital status: (Ref = divorced)**		
Long-term relationship	0.01 (0.11)	0.05 (0.12)
Married	−0.14 (0.12)	−0.17 (0.12)
Separated	−0.15 (0.11)	−0.24 (0.11)*
Single	0.07 (0.07)	0.02 (0.08)
Unmarried	−0.002 (0.11)	−0.05 (0.11)
Widowed	0.16 (0.15)	0.16 (0.15)
**First Nations: (Ref = no)**		
Yes	−0.03 (0.04)	−0.09 (0.05)
**Country of birth: (Ref = Australia)**		
Outside Australia	0.13 (0.05)**	0.19 (0.07)**

Abbreviation: SE, standard error; Ref, reference group.

The intercepts coefficient for univariable model (in order from top to bottom of the table): 3.66, 3.54, 3.49, 3.68, 3.57, 3.68 and 3.64.

The intercept coefficient for multivariable model is 3.49.

*p-value <.05.

**p-value <.01.

**Table 6.** Univariable and multivariable of linear mixed model of the domain ‘Willingness to ask for help’ of RAS

	Univariable model	Multivariable model
	Coefficients (SE)	Coefficients (SE)
**Age**	0.003 (0.001)**	0.006 (0.002)**
**Sex: (Ref = female)**		
Male	0.09 (0.03)**	1.09 (0.27)**
**Gender identity: (Ref = female)**		
Male	0.11 (0.04)**	−0.98 (0.28)**
Non-binary	−0.43 (0.18)*	−0.30 (0.20)
Transgender	0.27 (0.31)	0.05 (0.43)
**LGBTI: (Ref = no)**		
Yes	−0.33 (0.07)**	−0.31 (0.09)**
**Marital status: (Ref = divorced)**		
Long-term relationship	−0.07 (0.10)	0.10 (0.11)
Married	−0.18 (0.11)	−0.18 (0.11)
Separated	−0.21 (0.10)*	−0.21 (0.10)*
Single	−0.04 (0.06)	0.01 (0.07)
Unmarried	0.001 (0.10)	0.03 (0.10)
Widowed	−0.05 (0.14)	−0.04 (0.13)
**First Nations: (Ref = no)**		
Yes	0.04 (0.04)	−0.07 (0.05)
**Country of birth: (Ref = Australia)**		
Outside Australia	0.12 (0.05)**	0.17 (0.06)**

Abbreviation: SE, standard error; Ref, reference group.

The intercepts coefficient for univariable model (in order from top to bottom of the table): 3.81, 3.90, 3.88, 3.96, 3.98, 3.91 and 3.94.

The intercept coefficient for multivariable model is 3.70.

*p-value <.05.

**p-value <.01.

**Table 7.** Univariable and multivariable of linear mixed model of the domain ‘Goal and success orientation’ of RAS

	Univariable model	Multivariable model
	Coefficients (SE)	Coefficients (SE)
**Age**	−0.003 (0.001)*	−0.001 (0.002)
**Sex: (Ref = female)**		
Male	0.10 (0.03)**	0.62 (0.29)*
**Gender identity: (Ref = female)**		
Male	0.12 (0.04)**	−0.50 (0.29)
Non-binary	−0.36 (0.18)*	−0.32 (0.21)
Transgender	−0.13 (0.32)	−0.13 (0.44)
**LGBTI: (Ref = No)**		
Yes	−0.23 (0.08)**	−0.20 (0.10)*
**Marital status: (Ref = divorced)**		
Long-term relationship	0.09 (0.11)	0.04 (0.12)
Married	−0.07 (0.11)	−0.08 (0.12)
Separated	−0.11 (0.10)	−0.23 (0.11)*
Single	0.003 (0.07)	−0.06 (0.07)
Unmarried	−0.07 (0.10)	−0.11 (0.11)
Widowed	0.07 (0.14)	0.09 (0.14)
**First Nations: (Ref = no)**		
Yes	−0.02 (0.04)	−0.06 (0.05)
**Country of birth: (Ref = Australia)**		
Outside Australia	0.07 (0.05)	0.12 (0.07)

Abbreviation: SE, standard error; Ref, reference group.

The intercepts coefficient for univariable model (in order from top to bottom of the table): 3.97, 3.81, 3.76, 3.87, 3.83, 3.88 and 3.85.

The intercept coefficient for multivariable model is 3.91.

*p-value <.05.

**p-value <.01.

**Table 8.** Univariable and multivariable of linear mixed model of the domain ‘Reliance on others’ of RAS

	Univariable model	Multivariable model
	Coefficients (SE)	Coefficients (SE)
**Age**	−0.001 (0.001)	0.0006 (0.001)
**Sex: (Ref = female)**		
Male	0.07 (0.03)*	0.58 (0.26)*
**Gender identity: (Ref = female)**		
Male	0.07 (0.03)*	−0.50 (0.26)
Non-binary	−0.16 (0.16)	−0.16 (0.19)
Transgender	0.36 (0.29)	0.12 (0.40)
**LGBTI: (Ref = no)**		
Yes	−0.11 (0.07)	−0.17 (0.09)
**Marital status: (Ref = divorced)**		
Long-term relationship	0.08 (0.10)	0.11 (0.11)
Married	−0.07 (0.10)	−0.05 (0.11)
Separated	−0.11 (0.09)	−0.15 (0.10)
Single	0.02 (0.06)	−0.002 (0.07)
Unmarried	−0.09 (0.09)	−0.10 (0.10)
Widowed	−0.06 (0.13)	−0.05 (0.13)
**First Nations: (Ref = no)**		
Yes	0.03 (0.03)	0.007 (0.04)
**Country of birth: (Ref = Australia)**		
Outside Australia	0.04 (0.04)	0.05 (0.06)

Abbreviation: SE, standard error; Ref, reference group.

The intercepts coefficient for univariable model (in order from top to bottom of the table): 4.02, 3.93, 3.90, 3.99, 3.93, 3.96 and 3.89.

The intercept coefficient for multivariable model is 3.89.

*p-value <.05.

**p-value <.01.

**Table 9.** Univariable and multivariable of linear mixed model of the domain ‘No domination by symptoms’ of RAS

	Univariable model	Multivariable model
	Coefficients (SE)	Coefficients (SE)
**Age**	0.001 (0.001)	0.004 (0.002)
**Sex: (Ref = female)**		
Male	0.19 (0.04)**	0.51 (0.37)
**Gender identity: (Ref = female)**		
Male	0.25 (0.05)**	−0.26 (0.37)
Non-binary	−0.91 (0.24)**	−0.62 (0.28)*
Transgender	−0.15 (0.41)	−0.20 (0.56)
**LGBTI: (Ref = no)**		
Yes	−0.55 (0.10)*	−0.40 (0.12)**
**Marital status: (Ref = divorced)**		
Long-term relationship	−0.03 (0.14)	−0.009 (0.15)
Married	−0.11 (0.14)	−0.07 (0.15)
Separated	−0.05 (0.13)	−0.03 (0.14)
Single	0.10 (0.08)	−0.10 (0.09)
Unmarried	0.10 (0.13)	0.07 (0.14)
Widowed	0.25 (0.18)	0.25 (0.19)
**First Nations: (Ref = no)**		
Yes	−0.02 (0.05)	−0.08 (0.06)
**Country of birth: (Ref = Australia)**		
Outside Australia	0.10 (0.06)	0.10 (0.08)

Abbreviation: SE, standard error; Ref, reference group.

The intercepts coefficient for univariable model (in order from top to bottom of the table): 3.37, 3.32, 3.25, 3.45, 3.30, 3.44 and 3.41.

The intercept coefficient for multivariable model is 3.08.

*p-value <.05.

**p-value <.01.

The OR for male sex was 2.97 (95% CI: 1.75-5.05) in ‘willingness to ask for help’, 1.86 (95% CI: 1.05-3.28) in ‘goal and success orientation’, 1.82 (95% CI: 1.012-3.28) in ‘personal confidence and hope’ and 1.79 (95% CI: 1.07-1) in ‘reliance on others’. Overall, the OR for LGBTI were around 0.67 to 0.82 in four of the RAS domains as tabulated in Table 10.

**Table 10.** Odds ratios of statistically significant demographic characteristics (*p* < .05) for the RAS domains

	Demographic characteristics						
RAS domains, OR (95% CI)	Age	Male sex	Male gender identity	Non-binary gender identity	LGBTI	Born Outside Australia	Separated
Personal confidence and hope		1.82 (1.012-3.28)			0.71 (0.59-0.87)	1.21 (1.05-1.39)	0.79 (0.63-0.98)
Willingness to ask for help	0.994 (0.99-1)	2.97 (1.75-5.05)	0.38 (0.22-0.65)		0.73 (0.61-0.87)	1.19 (1.05-1.33)	0.81 (0.67-0.99)
Goal and success orientation		1.86 (1.05-3.28)			0.82 (0.67-1)		0.79 (0.64-0.99)
Reliance on others		1.79 (1.07-1)					
No domination by symptoms				0.54 (0.31-0.934)	0.67 (0.53-0.85)		

Abbreviation: OR, odds ratio; CI, confidence interval.

## Discussion

The time series for CANSAS and RAS follow-ups overtime were not affected by the seasonal and random components. The trend in the proportion of unmet needs of CANSAS reduced over time. The trend of RAS scores in all domains maintained and improved overtime consistently above scores of three. These trends of improvements indicate the effectiveness of HASI program in reducing the unmet needs and improve and facilitate the recovery of individuals with severe mental illness. Our discussion focuses on characteristics that have *p*-value of less than 0.01 rather than 0.05 due to the multiple tests performed.

## CANSAS

The younger age when first engaged in the service was associated with higher unmet needs in ‘accommodation’, ‘money’ and ‘benefit’ domains. However, older age was associated with higher unmet needs of ‘digital communication’ domain. However, the strength of association is rather weak with OR nearing one indicating that each year younger in age has minimal effect towards CANSAS unmet needs and RAS scores. Hence, the risk estimation between ‘younger’ and ‘older’ is only meaningful when a large age gap is compared. Nevertheless, we could assume that all three unmet needs found in younger people were interconnected by ‘money’. If they were not qualified in getting ‘benefit’ such as social welfare payment, they would have not had enough money to afford accommodation. HASI program is meant to provide accommodation support to reduce mental health related hospital admissions. With one unmet need resolved, and with the help of the support worker to apply the social welfare payment or able to focus on a job, these three unmet needs will be resolved over time. The older age seems to require more digital technology support. Hence, peer support worker could be more attentive to these needs by providing more support in using digital technology.

Male sex was associated with increased unmet needs in ‘sexual expression’ and ‘food’ domains. As such, additional counsel support on relationship may help in the male’s sexual expression unmet needs. Peer support worker could also support the budgeting of available money for food to resolve such unmet need in males. People who identified themselves as non-binary was associated with higher unmet needs of ‘company’ domain and as transgender was associated with higher unmet needs in ‘self-care’ and ‘food’ domains. The marital status with long-term relationship and being married were associated with less unmet needs in intimate relationship. People who identified themselves as First Nations were associated with higher unmet needs in ‘psychotic symptoms’, ‘safety to others’ and ‘transport’ domains. Thus, more specialised psychological care could be introduced alongside with peer support to further improve these ‘psychotic symptoms’ and ‘safety to others’ unmet needs. Peer support could also be more sensitive to and identify such needs as the priority by helping people who identified themselves as First Nations to obtain early appointment with psychologist or psychiatrist. This may further reduce hospitalisation. As most people of First Nations live in regional and rural areas, the unmet need of transportation was explained by the lack of public transport in those areas. Private transport is therefore needed to support the people of First Nations.

To our knowledge, no study specifically examined the demographic characteristics against each CANSAS domain. Nevertheless, recent systematic review findings indicate the age was associated with the unmet needs and male was associated with higher total unmet needs.^12^ Ethic minority was also associated with unmet needs but has conflicting results from two studies.^13,14^ The unmet needs of individuals with severe mental illness with aforementioned demographics have been broadly explored in literature. Unmet needs in sexual expression, except bipolar disorder, often associated with adverse drug effects, internalised and structural stigmatisation and complications with conditions.^15^ In our study, the association found could help support workers to prioritise the unmet needs of the people with certain characteristics, such as the First Nations and males in the HASI program.

## RAS

In ‘personal confidence and hope’ domain, people who identified themselves as LGBTI were associated with reduced RAS score but as born outside Australia, it was associated with increase RAS score. In ‘willingness to ask for help’, people who identified themselves as male gender and LGBTI were associated with reduced RAS score as opposed to older age, male sex and being born outside Australia, which were associated with an increase in RAS score.

Male sex was associated with an increase in RAS score but people who identified themselves as LGBTI and being separated were associated with reduced RAS score in ‘goal and success orientation’ domain. In ‘reliance on others’, male sex was associated with an increase in RAS score. People who identified themselves as LGBTI and non-binary gender identity was associated with reduced RAS score in domain ‘no domination by symptoms’.

To our knowledge, no study examined the relationship between demographic characteristics with the RAS domains. Despite the paucity of comparable study, it is evident that LGBTI individuals have more unmet health needs which may be associated with lower RAS score.^16,17^ In our study, demographic characteristics associated with the change in RAS score provide useful information for practice. For example, prioritising people who identified themselves as LGBTI in HASI program may improve the RAS scores in all domains because LGBTI was found to have reduced RAS scores in all domains.

## Limitations

Due to the declining number of assessments completed over time, missing data in some of the variables and the refusal to answer the outcome tools could affect the statistical model validity thereby reducing the likelihood of statistical significance. The selective loss to follow-up suggests potential selection bias which could threaten the validity of the findings. Despite such limitations, number of characteristics was found to be associated with both CANSAS and RAS outcomes.

Factors not examined in our study were education level and socio-economic status which may have influence in our study findings.^12^ Further study on these factors is needed to examine how these interact with demographic characteristics. More studies are also needed to substantiate our findings as this is the first finding in Australia that may not be generalisable to population with severe mental illness outside the country.

The validity findings of this study could be affected due to multiple supports received beyond Flourish Australia. Often, people with complex and severe mental health issue received more than one support in their recovery journey. Our study could not assess if there is additional support received apart from Flourish Australia. However, we do not believe that it is a major shortcoming as these account for only a few people who may need support other than HASI program. Hence, there should not be duplication of HASI program support and the grant funders, that is, NSW government, monitors each individual to ensure they only receive from one provider. Further study should account for this factor to validate the findings. Qualitative studies can reveal explanation on the association found between the demographic characteristics and CANSAS and with RAS.

## Conclusion

HASI program engagement is associated with an overall reduction of unmet needs and improvement of the recovery of individuals with severe mental illness. Age, sex, gender, LGBTI, First Nations and country of birth were associated with changes in the CANSAS and RAS outcomes. This is indicative that these demographic variables could aid in the prioritisation of HASI program services across Australia.

## Supplemental Material

Supplemental Material - Demographic and mental health characteristics of individuals in the NSW housing and accommodation support initiative (HASI), community living supports and HASI plus Supplemental material for Demographic and mental health characteristics of individuals in the NSW housing and accommodation support initiative (HASI), community living supports and HASI plus by Gary KK Low, Jason Li, Emily Hielscher, Veronica Sheanoda, Sumathi Govindasamy and Fadzi Marasha in Australasian Psychiatry
