# Supplementary material for: Demographic and mental health characteristics of individuals in the NSW Housing and Accommodation Support Initiative (HASI), Community Living Supports and HASI Plus
Source: Australas Psychiatry. 2025 Jan 30;33(3):493–504. doi: 10.1177/10398562251316431 (PMC12138147; doi:10.1177/10398562251316431)
Supplement: Supplemental Material - Demographic and mental health characteristics of individuals in the NSW housing and accommodation support initiative (HASI), community living supports and HASI plus [file sj-pdf-1-apy-10.1177_10398562251316431.pdf]

S1: Proportion of unmet needs and total needs in each CANSAS review

	Median	Q1	Q3	Total Needs	Total Needs (Q1)	Total Needs (Q3)
1	33.333333	12.500000	60.000000	9.0	5.00	13.00
2	26.666667	5.882353	50.000000	8.0	5.00	13.00
3	25.000000	0.000000	50.000000	8.0	5.00	13.00
4	22.222222	0.000000	50.000000	8.0	5.00	13.00
5	20.000000	0.000000	46.764706	8.0	4.00	13.00
6	20.000000	0.000000	40.000000	8.0	4.00	13.00
7	17.647059	0.000000	38.461538	8.0	4.00	13.00
8	17.647059	0.000000	40.000000	7.0	4.00	12.00
9	16.666667	0.000000	40.000000	8.0	5.00	13.00
10	12.500000	0.000000	36.363636	7.0	4.00	12.00
11	18.198529	0.000000	39.615385	7.0	4.00	12.00
12	15.587045	0.000000	33.333333	7.0	4.00	12.00
13	14.285714	0.000000	33.333333	8.0	4.00	13.00
14	14.285714	0.000000	36.931818	8.0	4.00	13.00
15	10.000000	0.000000	33.333333	8.0	4.50	12.50
16	11.437908	0.000000	37.335526	8.0	4.75	13.00
17	10.000000	0.000000	33.333333	9.0	5.00	14.00
18	8.846154	0.000000	28.571429	9.0	5.00	14.00
19	7.692308	0.000000	29.411765	8.0	4.00	14.00
20	5.882353	0.000000	30.769231	7.0	3.00	13.00
21	9.090909	0.000000	32.812500	9.0	5.00	14.00
22	7.692308	0.000000	27.777778	9.0	4.00	15.00
23	6.904762	0.000000	24.305556	9.0	4.00	13.00
24	7.142857	0.000000	25.000000	10.0	6.00	14.00
25	6.250000	0.000000	33.333333	9.0	4.75	16.00
26	9.545455	0.000000	30.312500	9.0	5.50	15.00
27	12.698413	0.000000	25.000000	9.0	5.00	15.00
28	13.333333	0.000000	28.571429	9.0	7.00	15.00
29	11.111111	0.000000	33.333333	10.0	7.00	16.00
30	5.050505	0.000000	29.444444	12.0	7.00	16.00
31	3.125000	0.000000	22.549020	12.0	7.00	16.00
32	6.787330	0.000000	25.892857	11.0	6.00	15.00
33	13.333333	0.000000	30.769231	13.0	8.00	17.00
34	6.666667	0.000000	25.000000	10.0	5.00	15.00
35	0.000000	0.000000	15.789474	9.5	6.00	18.75
36	15.789474	0.000000	28.571429	10.0	5.00	17.00
37	6.349206	0.000000	13.095238	13.0	6.75	15.50
38	0.000000	0.000000	25.000000	8.0	5.00	16.00
39	5.397727	1.136364	8.705357	18.5	12.25	21.00
40	0.000000	0.000000	0.000000	13.0	8.00	17.00
41	0.000000	0.000000	2.500000	11.5	3.75	18.50
42	2.941176	0.000000	9.967320	13.0	7.00	17.25
43	2.941176	1.470588	4.411765	13.0	6.50	15.00
44	0.000000	0.000000	7.142857	15.0	11.00	16.00
45	12.500000	6.250000	18.750000	7.0	5.50	8.50
46	0.000000	0.000000	0.000000	5.0	5.00	5.00
47	0.000000	0.000000	0.000000	11.0	11.00	11.00
48	0.000000	0.000000	0.000000	13.0	13.00	13.00
49	0.000000	0.000000	0.000000	18.0	18.00	18.00

Abbreviation: Q1, first quartile; Q3, third quartile.

S2: Decomposition of additive time series for CANSAS review.

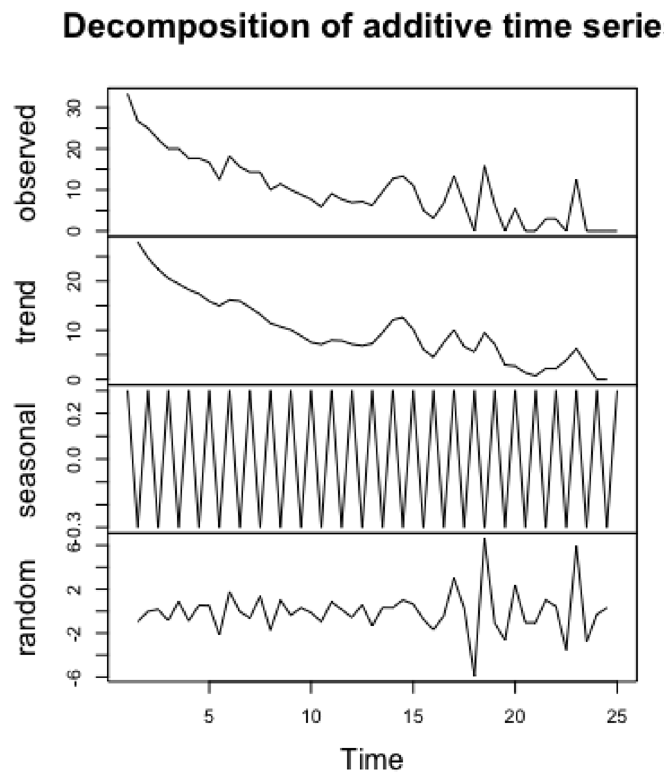

S3: Number of respondents in each review for CANSAS

Review No.	Frequency
1	2,007
2	1,652
3	1,376
4	1,152
5	981
6	840
7	730
8	630
9	559
10	510
11	464
12	415
13	374
14	328
15	289
16	265
17	241
18	210
19	188
20	160
21	137
22	122
23	104
24	89
25	76
26	69
27	59
28	51
29	46
30	38

Review No.	Frequency
31	32
32	26
33	22
34	21
35	14
36	13
37	10
38	9
39	6
40	5
41	4
42	4
43	4
44	4
45	2
46	1
47	1
48	1
49	1
50	1
51	1

S4: Univariable and multivariable generalised estimating equations of CANSAS "Accommodation" domain.

What kind of place do you live in?	Univariable model	Multivariable model
	Coefficients (SE)	Coefficients (SE)
Age	-0.02 (0.004)**	-0.02 (0.01)**
Sex: (Ref= Female)		
Male	-0.23 (0.09)*	0.32 (0.47)
Gender Identity: (Ref= Female)		
Male	-0.27 (0.11)*	-0.54 (0.48)
Non-binary	-0.01 (0.51)	-0.16 (0.60)
Transgender	0.53 (0.56)	0.42 (0.74)
LGBTI: (Ref=No)		
Yes	0.29 (0.22)	-0.09 (0.30)
Marital Status: (Ref=Divorced)		
Long-term relationship	0.15 (0.31)	-0.25 (0.36)
Married	-0.32 (0.39)	-0.41 (0.45)
Separated	0.67 (0.26)*	0.52 (0.30)
Single	0.01 (0.19)	-0.20 (0.23)
Unmarried	0.33 (0.29)	0.40 (0.32)
Widowed	-0.13 (0.48)	-0.15 (0.53)
First Nations: (Ref=No)		
Yes	0.43 (0.11)**	0.24 (0.15)
Country of Birth: (ref=Australia)		
Born Outside Australia	-0.43 (0.17)**	-0.05 (0.22)

Abbreviation: SE, standard error; Ref, Reference group.
The intercepts coefficients for univariable model (in order from top to bottom of the table): -0.63, -1.21, -1.27, -1.35, -1.42, -1.43 and -1.28.
The intercepts coefficients for multivariable model is -0.49.
*p-value<0.05
**p-value<0.01

S5: Univariable and multivariable generalised estimating equations of CANSAS "Food" domain.

Do you get enough to eat?	Univariable model	Multivariable model
	Coefficients (SE)	Coefficients (SE)
Age	-0.01 (0.003)*	-0.01 (0.01)
Sex: (Ref= Female)		
Male	-0.11 (0.09)	1.06 (0.47)*
Gender Identity: (Ref= Female)		
Male	-0.19 (0.11)	-1.18 (0.48)*
Non-binary	-0.83 (0.56)	-1.49 (0.70)*
Transgender	1.98 (0.73)**	2.29 (0.79)**
LGBTI: (Ref=No)		
Yes	0.24 (0.22)	0.20 (0.25)
Marital Status: (Ref=Divorced)		
Long-term relationship	-0.15 (0.34)	-0.56 (0.42)
Married	-0.01 (0.32)	-0.02 (0.33)
Separated	0.28 (0.27)	0.09 (0.31)
Single	-0.11 (0.18)	-0.37 (0.21)
Unmarried	-0.09 (0.30)	-0.32 (0.32)
Widowed	0.71 (0.39)	0.61 (0.38)
First Nations: (Ref=No)		
Yes	0.35 (0.11)**	0.38 (0.15)*

Country of Birth: (ref=Australia)		
Born Outside Australia	-0.14 (0.15)	-0.14 (0.20)

Abbreviation: SE, standard error; Ref, Reference group.
The intercepts coefficients for univariable model (in order from top to bottom of the table): -1.04, 1.35, -1.45, -1.43, -1.43, -1.50 and -1.41.
The intercepts coefficients for multivariable model is -0.96.
*p-value<0.05
**p-value<0.01

S6: Univariable and multivariable generalised estimating equations of CANSAS “Looking after the home” domain.

Are you able to look after your home?	Univariable model	Multivariable model
	Coefficients (SE)	Coefficients (SE)
Age	-0.01 (0.003)	-0.004 (0.004)
Sex: (Ref= Female)		
Male	-0.34 (0.08)**	0.20 (0.59)
Gender Identity: (Ref= Female)		
Male	-0.38 (0.09)**	-0.47 (0.59)
Non-binary	1.31 (0.48)**	1.12 (0.58)
Transgender	0.61 (0.60)	0.89 (0.81)
LGBTI: (Ref=No)		
Yes	0.54 (0.20)**	0.16 (0.27)
Marital Status: (Ref=Divorced)		
Long-term relationship	0.36 (0.25)	0.15 (0.28)
Married	0.23 (0.28)	0.09 (0.28)
Separated	0.33 (0.25)	0.14 (0.29)
Single	-0.22 (0.15)	-0.43 (0.17)*
Unmarried	-0.05 (0.24)	-0.15 (0.27)
Widowed	0.10 (0.35)	0.01 (0.37)
First Nations: (Ref=No)		
Yes	0.21 (0.10)*	0.19 (0.13)
Country of Birth: (ref=Australia)		
Born Outside Australia	-0.53 (0.14)**	-0.56 (0.19)**

Abbreviation: SE, standard error; Ref, Reference group.
The intercepts coefficients for univariable model (in order from top to bottom of the table): -0.77, -0.81, -0.86, -1.02, -0.88, -1.03 and -0.94.
The intercepts coefficients for multivariable model is -0.43.
*p-value<0.05
**p-value<0.01

S7: Univariable and multivariable generalised estimating equations of CANSAS “Self-care” domain.

Do you have problems keeping clean and tidy?	Univariable model	Multivariable model
	Coefficients (SE)	Coefficients (SE)
Age	-0.01 (0.003)*	-0.004 (0.004)
Sex: (Ref= Female)		
Male	-0.22 (0.09)*	-0.15 (0.32)
Gender Identity: (Ref= Female)		
Male	-0.24 (0.11)*	-0.06 (0.32)
Non-binary	1.31 (0.40)**	0.85 (0.48)
Transgender	1.57 (0.35)**	1.55 (0.48)**

LGBTI: (Ref=No)		
Yes	0.67 (0.18)**	0.43 (0.25)
Marital Status: (Ref=Divorced)		
Long-term relationship	0.35 (0.31)	0.25 (0.34)
Married	0.56 (0.28)*	0.55 (0.28)
Separated	-0.08 (0.28)	-0.13 (0.30)
Single	-0.12 (0.18)	-0.22 (0.21)
Unmarried	0.22 (0.27)	0.11 (0.30)
Widowed	0.36 (0.39)	0.33 (0.44)
First Nations: (Ref=No)		
Yes	0.15 (0.11)	0.05 (0.14)
Country of Birth: (ref=Australia)		
Born Outside Australia	-0.47 (0.16)**	-0.53 (0.21)*

Abbreviation: SE, standard error; Ref, Reference group.

The intercepts coefficients for univariable model (in order from top to bottom of the table): -0.89, -1.10, -1.15, -1.25, -1.19, -1.24 and -1.17.

The intercepts coefficients for multivariable model is -0.84.

*p-value<0.05

**p-value<0.01

S8: Univariable and multivariable generalised estimating equations of CANSAS "Daytime activities" domain.

How do you spend your day?	Univariable model	Multivariable model
	Coefficients (SE)	Coefficients (SE)
Age	-0.01 (0.003)**	-0.01 (0.003)*
Sex: (Ref= Female)		
Male	-0.07 (0.07)	0.40 (0.61)
Gender Identity: (Ref= Female)		
Male	-0.20 (0.08)*	-0.61 (0.61)
Non-binary	0.64 (0.43)	0.48 (0.49)
Transgender	0.78 (0.68)	0.64 (0.84)
LGBTI: (Ref=No)		
Yes	0.08 (0.18)	-0.12 (0.26)
Marital Status: (Ref=Divorced)		
Long-term relationship	0.42 (0.24)	0.35 (0.25)
Married	0.41 (0.24)	0.41 (0.25)
Separated	0.39 (0.21)	0.33 (0.23)
Single	0.22 (0.14)	0.12 (0.17)
Unmarried	0.25 (0.20)	0.20 (0.21)
Widowed	0.08 (0.29)	0.10 (0.29)
First Nations: (Ref=No)		
Yes	0.29 (0.09)**	0.19 (0.11)
Country of Birth: (ref=Australia)		
Born Outside Australia	-0.39 (0.11)**	-0.31 (0.15)*

Abbreviation: SE, standard error; Ref, Reference group.

The intercepts coefficients for univariable model (in order from top to bottom of the table): 0.23, -0.31, -0.42, -0.36, -0.68, -0.41 and -0.31.

The intercepts coefficients for multivariable model is -0.24.

*p-value<0.05

**p-value<0.01

S9: Univariable and multivariable generalised estimating equations of CANSAS "Physical health" domain.

How well do you feel physically?	Univariable model Coefficients (SE)	Multivariable model Coefficients (SE)
Age	-0.01 (0.003)**	-0.01 (0.003)
Sex: (Ref= Female)		
Male	-0.23 (0.07)**	0.70 (0.58)
Gender Identity: (Ref= Female)		
Male	-0.34 (0.08)**	-1.03 (0.59)
Non-binary	0.98 (0.41)*	0.46 (0.45)
Transgender	-0.14 (0.49)	-0.58 (0.56)
LGBTI: (Ref=No)		
Yes	0.41 (0.16)**	0.25 (0.21)
Marital Status: (Ref=Divorced)		
Long-term relationship	0.08 (0.24)	0.02 (0.26)
Married	0.18 (0.25)	0.16 (0.27)
Separated	0.27 (0.20)	0.24 (0.24)
Single	-0.05 (0.14)	-0.11 (0.16)
Unmarried	0.05 (0.22)	-0.03 (0.24)
Widowed	0.22 (0.29)	0.22 (0.29)
First Nations: (Ref=No)		
Yes	0.24 (0.09)**	0.17 (0.11)
Country of Birth: (ref=Australia)		
Born Outside Australia	-0.15 (0.11)	-0.17 (0.14)

Abbreviation: SE, standard error; Ref, Reference group.
The intercepts coefficients for univariable model (in order from top to bottom of the table): -0.29, -0.64, -0.66, -0.79, -0.79, -0.81 and -0.75.
The intercepts coefficients for multivariable model is -0.37.
*p-value<0.05
**p-value<0.01

S10: Univariable and multivariable generalised estimating equations of CANSAS "Psychotic symptoms" domain.

Do you ever hear voices or have problems with your thoughts?	Univariable model	Multivariable model
	Coefficients (SE)	Coefficients (SE)
Age	-0.01 (0.003)**	-0.01 (0.01)
Sex: (Ref= Female)		
Male	-0.23 (0.07)**	0.29 (0.79)
Gender Identity: (Ref= Female)		
Male	-0.34 (0.08)**	-0.68 (0.79)
Non-binary	0.98 (0.41)*	0.11 (0.65)
Transgender	-0.14 (0.49)	-0.83 (1.23)
LGBTI: (Ref=No)		
Yes	0.41 (0.16)**	0.14 (0.27)
Marital Status: (Ref=Divorced)		
Long-term relationship	0.08 (0.24)	0.59 (0.32)
Married	0.18 (0.25)	0.01 (0.41)
Separated	0.27 (0.20)	0.46 (0.27)
Single	-0.05 (0.14)	0.08 (0.18)
Unmarried	0.05 (0.22)	-0.18 (0.30)

Widowed	0.22 (0.29)	0.18 (0.40)
First Nations: (Ref=No)		
Yes	0.24 (0.09)**	0.44 (0.14)**
Country of Birth: (ref=Australia)		
Born Outside Australia	-0.15 (0.11)	-0.02 (0.19)

Abbreviation: SE, standard error; Ref, Reference group.
The intercepts coefficients for univariable model (in order from top to bottom of the table): -0.29, -0.64, -0.66, -0.79, -0.79, -0.81 and -0.75.
The intercepts coefficients for multivariable model is -0.37.
*p-value<0.05
**p-value<0.01

S11: Univariable and multivariable generalised estimating equations of CANSAS "Information on condition and treatment" domain.

Have you been given clear information about your medication?	Univariable model	Multivariable model
	Coefficients (SE)	Coefficients (SE)
Age	-0.01 (0.003)*	-0.01 (0.004)
Sex: (Ref= Female)		
Male	-0.27 (0.09)**	-0.11 (0.40)
Gender Identity: (Ref= Female)		
Male	-0.24 (0.11)*	-0.17 (0.41)
Non-binary	0.38 (0.43)	0.14 (0.55)
Transgender	0.56 (0.38)	0.89 (0.53)
LGBTI: (Ref=No)		
Yes	0.21 (0.21)	0.09 (0.30)
Marital Status: (Ref=Divorced)		
Long-term relationship	0.28 (0.33)	0.04 (0.36)
Married	0.20 (0.33)	0.02 (0.37)
Separated	0.38 (0.28)	0.49 (0.29)
Single	0.13 (0.20)	0.11 (0.21)
Unmarried	0.16 (0.31)	0.11 (0.35)
Widowed	0.39 (0.41)	0.48 (0.39)
First Nations: (Ref=No)		
Yes	0.36 (0.11)**	0.25 (0.13)
Country of Birth: (ref=Australia)		
Born Outside Australia	-0.13 (0.14)	-0.18 (0.19)

Abbreviation: SE, standard error; Ref, Reference group.
The intercepts coefficients for univariable model (in order from top to bottom of the table): -1.13, -1.35, -1.50, -1.50, -1.71, -1.57 and -1.48.
The intercepts coefficients for multivariable model is -1.29.
*p-value<0.05
**p-value<0.01

S12: Univariable and multivariable generalised estimating equations of CANSAS "Psychological distress" domain.

Have you recently felt very sad or low?	Univariable model	Multivariable model
	Coefficients (SE)	Coefficients (SE)
Age	-0.002 (0.002)	-0.01 (0.003)
Sex: (Ref= Female)		
Male	-0.32 (0.07)**	0.30 (0.67)

Gender Identity: (Ref= Female)		
Male	-0.42 (0.09)**	-0.71 (0.67)
Non-binary	0.76 (0.47)	0.33 (0.52)
Transgender	-0.41 (0.84)	-0.90 (1.10)
LGBTI: (Ref=No)		
Yes	0.43 (0.19)*	0.30 (0.24)
Marital Status: (Ref=Divorced)		
Long-term relationship	-0.04 (0.23)	-0.07 (0.25)
Married	0.004 (0.26)	0.02 (0.28)
Separated	0.40 (0.20)	0.33 (0.23)
Single	-0.16 (0.14)	-0.14 (0.16)
Unmarried	-0.08 (0.23)	-0.002 (0.24)
Widowed	0.09 (0.33)	0.13 (0.35)
First Nations: (Ref=No)		
Yes	0.23 (0.09)*	0.24 (0.12)*
Country of Birth: (ref=Australia)		
Born Outside Australia	-0.04 (0.12)	0.06 (0.16)

Abbreviation: SE, standard error; Ref, Reference group.
The intercepts coefficients for univariable model (in order from top to bottom of the table): -0.62, -0.58, -0.58, -0.77, -0.67, -0.79 and -0.74.
The intercepts coefficients for multivariable model is -0.26.
*p-value<0.05
**p-value<0.01

S13: Univariable and multivariable generalised estimating equations of CANSAS "Safety to self" domain.

Do you ever have thoughts of harming yourself?	Univariable model	Multivariable model
	Coefficients (SE)	Coefficients (SE)
Age	-0.01 (0.004)*	-0.01 (0.006)
Sex: (Ref= Female)		
Male	-0.37 (0.11)**	-0.26 (0.50)
Gender Identity: (Ref= Female)		
Male	-0.48 (0.14)**	-0.24 (0.51)
Non-binary	0.73 (0.46)	0.82 (0.61)
Transgender	0.64 (0.78)	1.43 (0.82)
LGBTI: (Ref=No)		
Yes	0.19 (0.26)	-0.23 (0.40)
Marital Status: (Ref=Divorced)		
Long-term relationship	0.25 (0.41)	-0.05 (0.50)
Married	0.09 (0.44)	0.01 (0.50)
Separated	0.24 (0.36)	0.11 (0.41)
Single	0.03 (0.27)	0.02 (0.33)
Unmarried	0.07 (0.39)	0.07 (0.46)
Widowed	-0.18 (0.54)	-0.06 (0.58)
First Nations: (Ref=No)		
Yes	0.30 (0.14)*	0.15 (0.18)
Country of Birth: (ref=Australia)		
Born Outside Australia	-0.24 (0.19)	-0.12 (0.27)

Abbreviation: SE, standard error; Ref, Reference group.
The intercepts coefficients for univariable model (in order from top to bottom of the table): -1.09, -1.32, -1.47, -1.53, -1.67, -1.58 and -1.49.
The intercepts coefficients for multivariable model is -1.07.
*p-value<0.05
**p-value<0.01

S14: Univariable and multivariable generalised estimating equations of CANSAS "Safety to others" domain.

Do you think you could be a danger to other people's safety?	Univariable model	Multivariable model
	Coefficients (SE)	Coefficients (SE)
Age	-0.03 (0.01)**	-0.02 (0.01)
Sex: (Ref= Female)		
Male	-0.17 (0.17)	0.35 (0.90)
Gender Identity: (Ref= Female)		
Male	-0.22 (0.22)	-0.58 (0.90)
Non-binary	-0.51 (0.98)	-1.22 (1.21)
Transgender	1.14 (1.27)	1.40 (1.57)
LGBTI: (Ref=No)		
Yes	-0.13 (0.58)	0.29 (0.71)
Marital Status: (Ref=Divorced)		
Long-term relationship	1.13 (0.51)*	0.80 (0.60)
Married	-0.14 (0.86)	0.02 (1.07)
Separated	-0.86 (0.71)	-0.67 (0.72)
Single	-0.06 (0.40)	-0.18 (0.45)
Unmarried	-0.16 (0.56)	-0.17 (0.65)
Widowed	-0.96 (1.07)	-0.71 (1.13)
First Nations: (Ref=No)		
Yes	0.94 (0.18)**	0.85 (0.25)**
Country of Birth: (ref=Australia)		
Born Outside Australia	-0.69 (0.32)*	-0.21 (0.51)

Abbreviation: SE, standard error; Ref, Reference group.
The intercepts coefficients for univariable model (in order from top to bottom of the table): -1.12, -2.11, -2.42, -2.19, -2.37, -2.47 and -2.14.
The intercepts coefficients for multivariable model is -1.86.
*p-value<0.05
**p-value<0.01

S15: Univariable and multivariable generalised estimating equations of CANSAS "Company" domain.

Are you happy with your social life?	Univariable model	Multivariable model
	Coefficients (SE)	Coefficients (SE)
Age	-0.01 (0.002)**	$-0.04x10^{3}$ (0.004)
Sex: (Ref= Female)		
Male	0.01 (0.07)	-0.31 (0.45)
Gender Identity: (Ref= Female)		
Male	-0.20 (0.07)*	0.07 (0.46)
Non-binary	1.41 (0.37)**	1.60 (0.48)**
Transgender	0.18 (0.52)	0.01 (0.59)
LGBTI: (Ref=No)		
Yes	0.12 (0.19)	-0.09 (0.26)
Marital Status: (Ref=Divorced)		
Long-term relationship	0.06 (0.24)	0.08 (0.27)
Married	-0.23 (0.30)	-0.23 (0.31)
Separated	-0.01 (0.21)	-0.14 (0.23)
Single	0.01 (0.14)	0.01 (0.16)
Unmarried	0.31 (0.24)	0.29 (0.27)
Widowed	0.36 (0.34)	0.40 (0.34)
First Nations: (Ref=No)		

Yes	-0.15 (0.09)	-0.12 (0.12)
Country of Birth: (ref=Australia)		
Born Outside Australia	-0.26 (0.11)*	-0.30 (0.16)

Abbreviation: SE, standard error; Ref, Reference group.

The intercepts coefficients for univariable model (in order from top to bottom of the table): -0.05, -0.38, -0.41, -0.39, -0.48, -0.35 and -0.35.

The intercepts coefficients for multivariable model is -0.37.

*p-value<0.05

**p-value<0.01

S16: Univariable and multivariable generalised estimating equations of CANSAS "Intimate relationships" domain.

Do you have a partner?	Univariable model	Multivariable model
	Coefficients (SE)	Coefficients (SE)
Age	0.01 (0.004)	0.01 (0.01)*
Sex: (Ref= Female)		
Male	0.54 (0.10)**	0.77 (0.60)
Gender Identity: (Ref= Female)		
Male	0.46 (0.12)**	-0.42 (0.60)
Non-binary	0.26 (0.62)	0.73 (0.65)
Transgender	0.32 (0.75)	0.74 (0.76)
LGBTI: (Ref=No)		
Yes	-0.17 (0.25)	-0.44 (0.32)
Marital Status: (Ref=Divorced)		
Long-term relationship	-1.42 (0.35)**	-1.25 (0.38)**
Married	-1.63 (0.38)**	-1.56 (0.43)**
Separated	-0.49 (0.31)	-0.60 (0.33)
Single	-0.17 (0.21)	-0.11 (0.24)
Unmarried	0.08 (0.33)	0.27 (0.37)
Widowed	0.18 (0.54)	0.14 (0.54)
First Nations: (Ref=No)		
Yes	-0.11 (0.13)	0.10 (0.16)
Country of Birth: (ref=Australia)		
Born Outside Australia	-0.14 (0.16)	-0.19 (0.22)

Abbreviation: SE, standard error; Ref, Reference group.

The intercepts coefficients for univariable model (in order from top to bottom of the table): 0.03, -0.08, -0.10, 0.23, 0.45, 0.24 and 0.24.

The intercepts coefficients for multivariable model is -0.34.

*p-value<0.05

**p-value<0.01

S17: Univariable and multivariable generalised estimating equations of CANSAS "Sexual expression" domain.

How is your sex life?	Univariable model	Multivariable model
	Coefficients (SE)	Coefficients (SE)

Age	0.01 (0.005)*	0.01 (0.01)*
Sex: (Ref= Female)		
Male	0.44 (0.12)**	1.11 (0.40)**
Gender Identity: (Ref= Female)		
Male	0.45 (0.14)**	-0.75 (0.41)
Non-binary	0.51 (0.59)	0.80 (0.62)
Transgender	-1.79 (0.93)	-1.47 (1.42)
LGBTI: (Ref=No)		
Yes	-0.15 (0.26)	-0.36 (0.34)
Marital Status: (Ref=Divorced)		
Long-term relationship	-0.60 (0.46)	-0.59 (0.49)
Married	-1.09 (0.48)*	-1.06 (0.53)*
Separated	-1.02 (0.41)*	-1.14 (0.45)*
Single	-0.20 (0.28)	-0.30 (0.31)
Unmarried	0.46 (0.42)	0.49 (0.47)
Widowed	-0.06 (0.52)	-0.21 (0.51)
First Nations: (Ref=No)		
Yes	-0.27 (0.16)	-0.34 (0.20)
Country of Birth: (ref=Australia)		
Born Outside Australia	-0.40 (0.19)*	-0.58 (0.28)*

Abbreviation: SE, standard error; Ref, Reference group.
The intercepts coefficients for univariable model (in order from top to bottom of the table): -0.66, -0.53, -0.64, -0.25, -0.12, -0.22 and -0.22.
The intercepts coefficients for multivariable model is -0.73.
*p-value<0.05
**p-value<0.01

S18: Univariable and multivariable generalised estimating equations of CANSAS "Digital communication" domain.

Do you have a phone and access to the internet?	Univariable model	Multivariable model
	Coefficients (SE)	Coefficients (SE)
Age	0.01 (0.004)	0.02 (0.01)**
Sex: (Ref= Female)		
Male	0.13 (0.11)	0.46 (0.71)
Gender Identity: (Ref= Female)		
Male	0.14 (0.13)	-0.38 (0.71)
Non-binary	-0.50 (0.77)	-0.20 (1.06)
Transgender	0.61 (0.75)	1.40 (1.05)
LGBTI: (Ref=No)		
Yes	-0.29 (0.33)	-0.28 (0.45)
Marital Status: (Ref=Divorced)		
Long-term relationship	0.42 (0.39)	0.34 (0.45)
Married	-0.16 (0.39)	-0.11 (0.42)
Separated	0.49 (0.35)	0.32 (0.40)
Single	0.20 (0.25)	0.32 (0.26)
Unmarried	0.46 (0.39)	0.40 (0.39)
Widowed	0.64 (0.39)	0.30 (0.42)
First Nations: (Ref=No)		
Yes	0.31 (0.14)*	0.27 (0.18)
Country of Birth: (ref=Australia)		
Born Outside Australia	-0.35 (0.18)	-0.61 (0.24)*

Abbreviation: SE, standard error; Ref, Reference group.
The intercepts coefficients for univariable model (in order from top to bottom of the table): -1.87, -1.66, -1.74, -1.58, -1.92, -1.65 and -1.56.
The intercepts coefficients for multivariable model is -2.91.
*p-value<0.05

**p-value<0.01

S19: Univariable and multivariable generalised estimating equations of CANSAS "Transport" domain.

How do you find using the bus, tram or train?	Univariable model	Multivariable model
	Coefficients (SE)	Coefficients (SE)
Age	-0.01 (0.003)**	-0.01 (0.004)
Sex: (Ref= Female)		
Male	-0.22 (0.10)	0.44 (0.35)
Gender Identity: (Ref= Female)		
Male	-0.15 (0.33)*	-0.63 (0.35)
Non-binary	0.15 (0.33)	-0.28 (0.43)
Transgender	0.74 (0.82)	-0.31 (1.32)
LGBTI: (Ref=No)		
Yes	0.02 (0.19)	0.20 (0.27)
Marital Status: (Ref=Divorced)		
Long-term relationship	0.37 (0.30)	0.17 (0.34)
Married	0.13 (0.30)	0.17 (0.32)
Separated	0.34 (0.27)	-0.01 (0.29)
Single	0.08 (0.17)	-0.15 (0.19)
Unmarried	0.57 (0.24)*	0.33 (0.26)
Widowed	0.41 (0.33)	0.35 (0.31)
First Nations: (Ref=No)		
Yes	0.42 (0.10)**	0.47 (0.14)**
Country of Birth: (ref=Australia)		
Born Outside Australia	-0.30 (0.13)*	-0.18 (0.18)

Abbreviation: SE, standard error; Ref, Reference group.
The intercepts coefficients for univariable model (in order from top to bottom of the table): -0.72, -1.07, -1.15, -1.14, -1.32, -1.22 and -1.10.
The intercepts coefficients for multivariable model is -0.88.
*p-value<0.05
**p-value<0.01

S20: Univariable and multivariable generalised estimating equations of CANSAS "Money" domain.

How do you find budgeting your money?	Univariable model	Multivariable model
	Coefficients (SE)	Coefficients (SE)
Age	-0.02 (0.003)**	-0.02 (0.004)**
Sex: (Ref= Female)		
Male	-1.07 (0.08)	0.75 (0.80)
Gender Identity: (Ref= Female)		
Male	-0.16 (0.10)	-0.95 (0.80)
Non-binary	0.17 (0.39)	-0.64 (0.46)
Transgender	0.14 (0.87)	-0.11 (1.07)
LGBTI: (Ref=No)		
Yes	0.17 (0.20)	0.36 (0.27)
Marital Status: (Ref=Divorced)		
Long-term relationship	0.26 (0.28)	0.02 (0.30)
Married	-0.18 (0.30)	-0.28 (0.33)
Separated	0.22 (0.25)	0.08 (0.28)
Single	0.04 (0.18)	-0.21 (0.22)

Unmarried	0.30 (0.26)	0.30 (0.29)
Widowed	-0.54 (0.39)	-0.40 (0.38)
First Nations: (Ref=No)		
Yes	0.21 (0.10)*	0.13 (0.14)
Country of Birth: (ref=Australia)		
Born Outside Australia	-0.06 (0.13)	-0.02 (0.19)

Abbreviation: SE, standard error; Ref, Reference group.
The intercepts coefficients for univariable model (in order from top to bottom of the table): -0.40, -0.03, -1.11, -1.11, -1.20, -1.14 and -1.09.
The intercepts coefficients for multivariable model is -0.08.
*p-value<0.05
**p-value<0.01

S21: Univariable and multivariable generalised estimating equations of CANSAS "Benefits" domain.

Are you getting all the money you are entitled to?	Univariable model	Multivariable model
	Coefficients (SE)	Coefficients (SE)
Age	-0.02 (0.004)**	-0.02 (0.006)**
Sex: (Ref= Female)		
Male	-0.06 (0.11)	0.70 (0.53)
Gender Identity: (Ref= Female)		
Male	-0.14 (0.13)	-0.79 (0.53)
Non-binary	0.43 (0.65)	-0.10 (0.80)
Transgender	1.64 (0.76)*	1.71 (1.13)
LGBTI: (Ref=No)		
Yes	0.32 (0.25)	0.10 (0.32)
Marital Status: (Ref=Divorced)		
Long-term relationship	0.22 (0.37)	-0.18 (0.40)
Married	0.16 (0.43)	0.02 (0.49)
Separated	0.62 (0.31)*	0.52 (0.32)
Single	0.20 (0.23)	-0.17 (0.25)
Unmarried	0.15 (0.34)	0.07 (0.34)
Widowed	0.77 (0.43)	0.80 (0.45)
First Nations: (Ref=No)		
Yes	-0.07 (0.13)	-0.15 (0.16)
Country of Birth: (ref=Australia)		
Born Outside Australia	-0.27 (0.20)	-0.22 (0.26)

Abbreviation: SE, standard error; Ref, Reference group.
The intercepts coefficients for univariable model (in order from top to bottom of the table): -1.07, -1.77, -1.80, -1.82, -2.07, -1.80 and -1.78.
The intercepts coefficients for multivariable model is -0.85.
*p-value<0.05
**p-value<0.01

S22: Decomposition of additive time series for RAS review on domain: "personal confidence and hope".

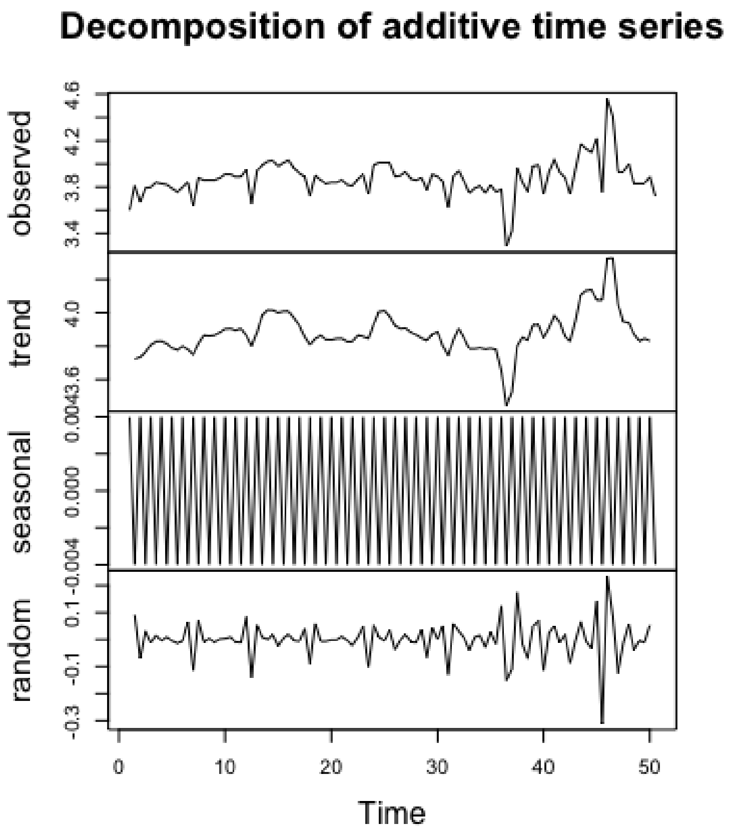

S23: Decomposition of additive time series for RAS review on domain: “willingness to ask for help”.

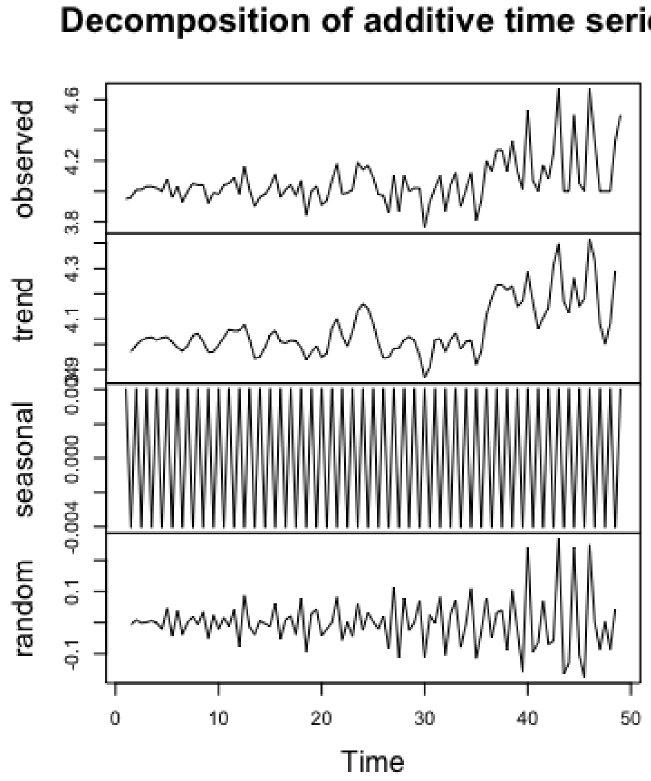

S24: Decomposition of additive time series for RAS review on domain: “goal and success orientation”.

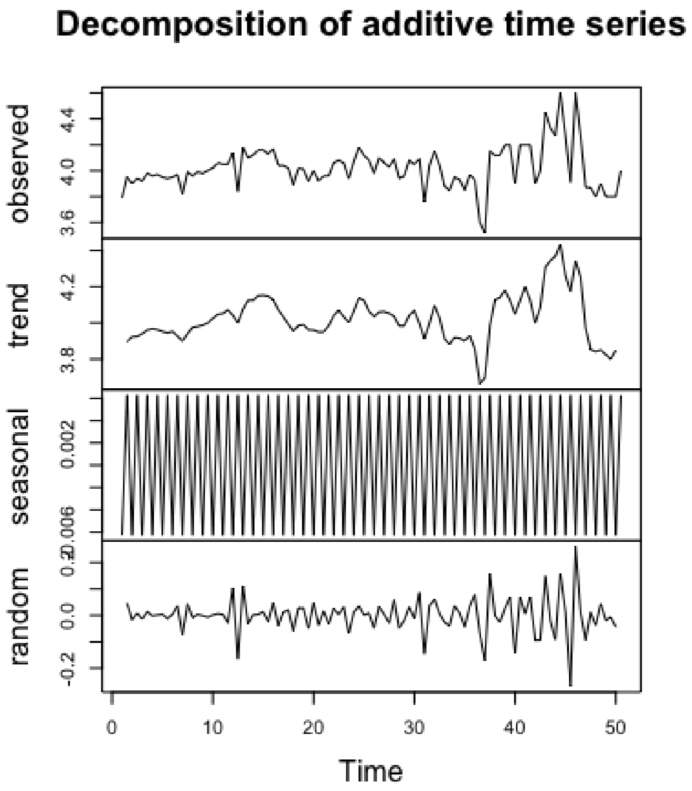

S25: Decomposition of additive time series for RAS review on domain: “reliance on others”.

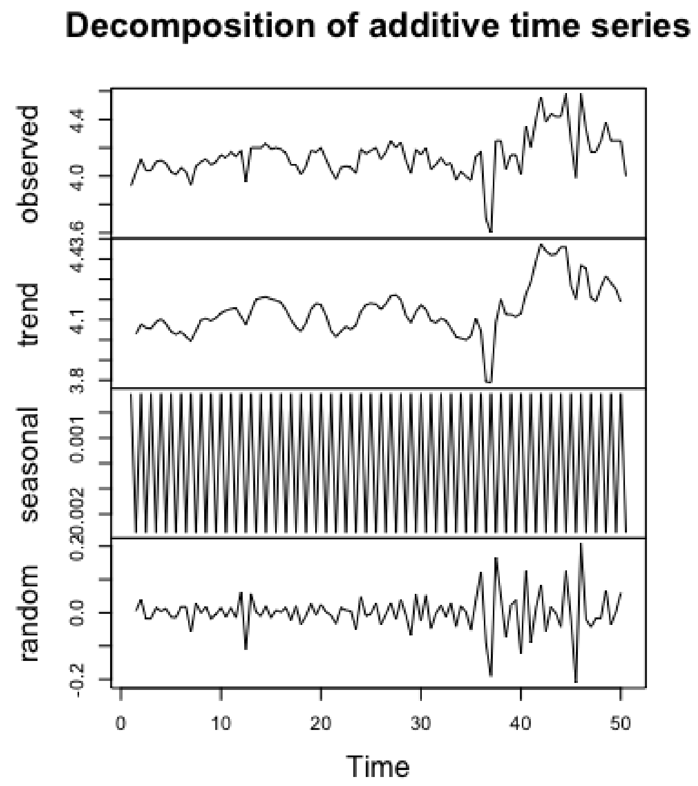

S26: Decomposition of additive time series for RAS review on domain: “no domination by symptoms”.

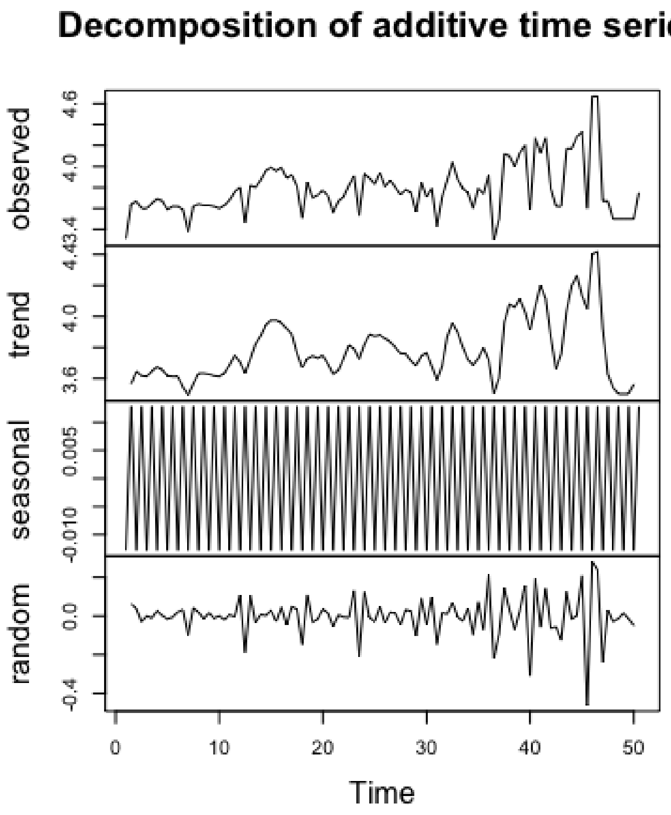

S27: Number of respondents in each review for RAS

Review No.	Frequency
1	2,350
2	1,427
3	1,193
4	1,031
5	875
6	801
7	684
8	631
9	563
10	504
11	453
12	428
13	388
14	369
15	340
16	316
17	291
18	272
19	253
20	232
21	216
22	198
23	186
24	174
25	166
26	156
27	148
28	140
29	127
30	119
31	107
32	97
33	88
34	83
35	74
36	70
37	65
38	63
39	61
40	57
41	53
42	52
43	50
44	50
45	49
46	46
47	42
48	41
49	38
50	37
51	34
52	32
53	29
54	26
55	24
56	23
57	21
58	19
59	16
60	16
61	15
62	15
63	15
64	15

Review No.	Frequency
65	13
66	11
67	8
68	8
69	8
70	8
71	7
72	6
73	5
74	5
75	5
76	5
77	5
78	5
79	5
80	5
81	5
82	5
83	4
84	4
85	4
86	3
87	3
88	3
89	3
90	3
91	3
92	3
93	3
94	2
95	2
96	2
97	2
98	2
99	2
100	2
101	1
102	1
103	1
104	1
105	1
106	1
107	1
108	1
109	1
110	1
111	1
112	1
113	1
114	1
115	1
116	1
117	1
118	1
119	1
120	1
121	1
122	1
123	1
124	1
125	1
126	1
127	1
128	1
129	1
130	1

Review No.	Frequency
131	1
132	1
133	1
134	1
135	1
136	1
137	1
138	1
139	1
